# Directed Evolution of Improved Zinc Finger Methyltransferases

**DOI:** 10.1371/journal.pone.0096931

**Published:** 2014-05-08

**Authors:** Brian Chaikind, Marc Ostermeier

**Affiliations:** 1 Chemistry-Biology Interface Graduate Program, Johns Hopkins University, Baltimore, Maryland, United States of America; 2 Department of Chemical and Biomolecular Engineering, Johns Hopkins University, Baltimore, Maryland, United States of America; Imperial College London, United Kingdom

## Abstract

The ability to target DNA methylation toward a single, user-designated CpG site *in vivo* may have wide applicability for basic biological and biomedical research. A tool for targeting methylation toward single sites could be used to study the effects of individual methylation events on transcription, protein recruitment to DNA, and the dynamics of such epigenetic alterations. Although various tools for directing methylation to promoters exist, none offers the ability to localize methylation solely to a single CpG site. In our ongoing research to create such a tool, we have pursued a strategy employing artificially bifurcated DNA methyltransferases; each methyltransferase fragment is fused to zinc finger proteins with affinity for sequences flanking a targeted CpG site for methylation. We sought to improve the targeting of these enzymes by reducing the methyltransferase activity at non-targeted sites while maintaining high levels of activity at a targeted site. Here we demonstrate an *in vitro* directed evolution selection strategy to improve methyltransferase specificity and use it to optimize an engineered zinc finger methyltransferase derived from M.SssI. The unusual restriction enzyme McrBC is a key component of this strategy and is used to select against methyltransferases that methylate multiple sites on a plasmid. This strategy allowed us to quickly identify mutants with high levels of methylation at the target site (up to ∼80%) and nearly unobservable levels of methylation at a off-target sites (<1%), as assessed in *E. coli*. We also demonstrate that replacing the zinc finger domains with new zinc fingers redirects the methylation to a new target CpG site flanked by the corresponding zinc finger binding sequences.

## Introduction

CpG methylation is one of the most extensively studied epigenetic modifications; it broadly regulates and maintains transcriptional repression. CpG methylation is involved in proper cellular differentiation, heterochromatin formation and in maintaining chromosomal stability [1]. Further, aberrant methylation patterns cause or are observed in numerous diseases. Imprinting defects lead to disorders such as Prader-Willi and Angelman syndromes [2]. Notably, global genomic hypomethylation and local hypermethylation of CpG islands (CGIs) commonly occur in cancer [3]. Though much has been learned about how methylation patterns are established and erased, the causes of aberrant methylation and the reestablishment of methylation patterns during development remain active areas of research. To study the effects and dynamics of DNA methylation, it would be generally useful to target methylation toward specific, user-defined sequences.

Several groups have engineered methyltransferases that direct methylation towards user-defined DNA sequences. The general strategy, pioneered by Xu and Bestor, involves fusion of a sequence specific DNA binding protein to a methyltransferase enzyme [4]. These constructs have been used to affect methylation, *in vitro*, in *E. coli,* and in cancer cell lines [5]–[10]. These directed methyltransferases have been shown to stably and heritably reduce the expression of *Sox2* and *Maspin* genes [11]. Siddique *et al.* demonstrated that targeting methylation towards the VEGF-A promoter significantly reduced gene expression in SKOV3 cells [12]. A recent review summarizes much of the literature on targeted methylation [13]. However, the engineered enzymes mentioned above methylate multiple CpG sites adjacent to the targeted DNA sequence. Despite the successes of these studies in biasing methylation to a particular region, only a few studies have focused on targeting methylation to single CpG sites [14]–[17].

Though methylation at single sites in eukaryotes is not believed to be the main means of epigenetic transcriptional silencing, multiple studies suggest single methylation events can alter the expression levels of select genes. *In vitro* methylation of a single CpG site within the *S1000A2* promoter on a reporter plasmid resulted in significant downregulation of gene expression, upon transfection, relative to an unmethylated, transfected control [18]. Methylated oligonucleotides targeting an intronic region of *peroxisomal membrane protein 4* (*PXMP4 or PMP24*) resulted in a single methylation mark on chromosomal DNA that downregulated gene expression relative to controls; this result corroborated differences observed between normal tissues and tumor cells [19]. Electromobility shift assays show that methylation at a single site impairs the binding of the genomic insulator CTCF [20].

In addition to studying effects on transcription, an engineered methyltransferase that specifically methylates a single site in a promoter would be generally useful for studying the effects of single aberrant methylation events on the propagation, maintenance, and correction of epigenetic marks. Finally, methyltransferases were recently engineered to more efficiently incorporate the transfer of unnatural alkyl groups donated by S-adenosylmethionine cofactor analogues [21]. This may make it possible to use targeted methyltransferases to site-specifically label DNA.

We desire a tool to assess the effects of single methylation events within the chromosome of human cell lines. As a first step, we describe significant progress made toward designing enzymes that target methylation at single CpG sites flanked by user-defined sequences in *E. coli.* The lack of endogenous CpG methyltransferases in *E. coli* facilitates the assessment target and off-target activities *in vivo*.

Our strategy for achieving single-site, targeted methylation is to make the assembly of a heterodimeric methyltransferase dependent on specific DNA sequences flanking a site to be methylated. To accomplish this task, we have employed naturally [16] or artificially split [17] DNA methyltransferases and altered these heterodimers to reduce their innate ability to reassemble into a functional enzyme. Reducing the ability of the fragments to self-assemble is necessary as we and others have shown that bifurcated methyltransferases are capable of unassisted reassembly into functional enzymes [22]–[25]. These reassembly-defective fragments are fused to zinc fingers, whose recognition sequences flank the targeted CpG site. The zinc finger domains bind to DNA, increasing the local concentration of the fused methyltransferase fragments over a targeted CpG site. Proper orientation of the methyltransferase fragment-zinc finger fusions at the target site primes the fragments for reassembly into a functional enzyme. The orientation of the fragments at the target site is affected by the topology of the fusions and the amino acid linker lengths connecting protein domains [17]. Optimization of these parameters, as well as the reduction of the affinity of fragments for each other and for DNA, reduces the enzyme’s non-specific activity and promotes enzymatic reassembly at the targeted CpG site [15]–[17].

We have previously demonstrated that, when fused to zinc finger proteins, a split version of M.SssI will bias methylation toward a targeted CpG site flanked by two cognate zinc finger binding sequences [17]. The DNA and amino acid sequence of this engineered protein is provided as Figure S1. The bifurcation point in M.SssI was chosen based on a CLUSTALW alignment to a site in a similarly engineered M.HhaI enzyme [17]. Monomeric M.SssI naturally methylates CpG sites [26]. Although the bifurcated M.SssI construct methylated the target site, it also methylated other M.SssI sites [17]. Site specific mutations Q147L or S317A in the M.SssI domain, introduced to reduce the enzyme’s DNA binding affinity and activity, reduced unwanted methylation at these other CpG sites [17]. We sought to reduce off-target methylation without affecting levels of methylation at the targeted site. Here we present a selection strategy to improve the targeting of methyltransferases toward new sites and have used this strategy to optimize our M.SssI fusion construct. We performed a negative selection against off-target methylation and a positive selection for methylation at a target site *in vitro*. This strategy allowed us to quickly identify variants with improved targeting ability and activity *in vivo*. We also demonstrate the modularity of our constructs by altering the zinc finger domains to redirect methylation toward a new target site.

## Materials and Methods

### Enzymes, Oligonucleotides and Bacterial Strains

Restriction enzymes, T4 ligase, T4 kinase, and Phusion High Fidelity PCR MMX were purchased from New England Biolabs (Ipswich, MA). BoxI was purchased from ThermoFisher Scientific (Waltham, MA). Platinum Pfx DNA polymerase was purchased from Life Technologies (Carlsbad, CA). PfuTurbo Cx Hotstart DNA polymerase was purchase from Agilent Technologies (Santa Clara, CA). Plasmid-Safe-ATP-dependent DNAse was purchased from Epicentre (Madison, WI). pDIMN8 and pAR plasmids have been previously described [16], [17]. All oligonucleotides and gBlocks were synthesized by Invitrogen (Carlsbad, CA) or Integrated DNA Technologies (Coralville, IA). Gel electrophoresis and PCR were preformed essentially as previously described [27]. Plasmids were isolated using QIAprep Spin Miniprep Kit (Qiagen, Valencia, CA). DNA fragments were purified from agarose gels using QIAquick Gel Extraction Kit (Qiagen, Valencia, CA) or PureLink Quick Gel Extraction Kit (Invitrogen, Carlsbad,CA, USA) and further concentrated using DNA Clean & Concentrator-5 (Zymo Research, Irvine, CA).

Escherichia coli K-12 strain ER2267 *[F proA^+^B^+^ lacI^q^ D(lacZ)M15 zzf::mini-Tn10 (Kan^R^)/D(argF-lacZ)U169 glnV44 e14^–^(McrA^–^) rfbD1? recA1 relA1? endA1 spoT1? thi-1 D(mcrC-mrr)114::IS10]* was acquired from New England Biolabs (Ipswich, MA) and was used in selections, methylation assays and cloning. NEB 5-alpha Competent *E. coli* (High Efficiency) *[fhuA2D(argF-lacZ)U169 phoA glnV44 φ80Δ(lacZ)M15 gyrA96 recA1 relA1 endA1 thi-1 hsdR17]* were also used for cloning and were purchased from New England Biolabs (Ipswich, MA).

### Plasmid Creation

pDIMN8 was used for library creation and testing of library variants [17]. pDIMN9 was constructed as follows for use in golden gate cloning. Plasmid pDIMN8 was altered by silently mutating a BsaI site in the Amp^R^ gene via pFunkel mutagenesis [28]. PCR, digestion and cloning removed a BbsI restriction site to create vector pDIMN9. Golden gate cloning was used to fuse new zinc finger proteins to methyltransferase fragments. For the creation of plasmids used in golden gate cloning, regions encoding zinc finger proteins were replaced with BbsI sites. pDIMN9 contained a M.SssI [1–272]-BbsI construct for the addition of zinc fingers to the N-terminal fragment. pAR contained BbsI-M.SssI [273–386] construct for the addition of new zinc fingers to the C-terminal fragments [16]. gBlocks encoding zinc fingers and BbsI sites were purchased from Integrated DNA Technologies. Golden gate cloning to fuse zinc finger-encoding gBlocks to the above plasmids was performed essentially as described [29]. Zinc finger CD54a was designed using the zinc finger tools website and previously identified zinc finger domains [30]–[32]. As previously described, plasmids containing genes encoding individual C-terminal and N-terminal zinc finger-fused proteins were digested with EcoRI and SpeI and ligated together, in order to place these genes on a single large plasmid for characterization in *E. coli* [16]. Site 1 and site 2 on this plasmid refer to previously described cloning sites on this large plasmid [17] and were used to construct the various target and non-target sites described in the study.

### Construction of Cassette Mutagenesis Library

**Figure 1:**
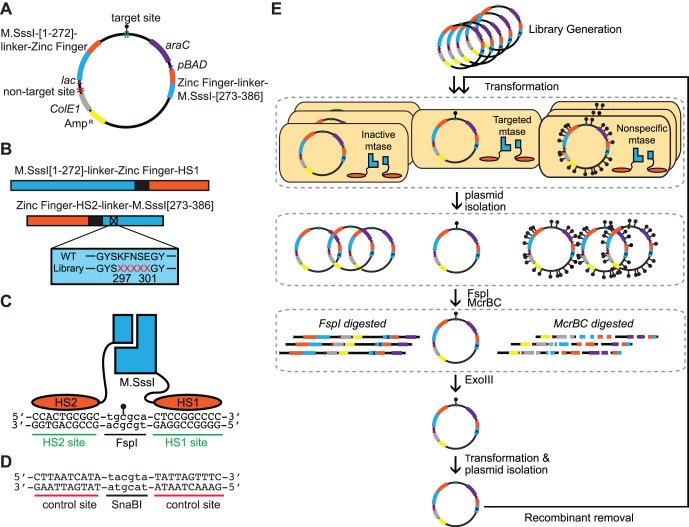
Schematics of the vector, library, proteins, and selection used in these experiments. (A) The vector used in selections. The vector encodes for both heterodimeric fragments fused to zinc fingers under the control of separate inducible arabinose (*pBAD*) and IPTG (*lac*) promoters, a target site, and the *araC* gene. (B) A schema of the zinc finger-fused, bifurcated M.SssI and the mutagenized codons used in library construction. Codons corresponding to residues 297–301 of M.SssI (located in the C-terminal fragment) were randomized. Numbering scheme is that of the wildtype M.SssI. (C) An assembled zinc finger-fused heterodimeric M.SssI methyltransferase assembled at the target site. The target site consists of an internal CpG site nested within an FspI restriction site, and flanked by HS1 and HS2 recognition sequences. (D) The non-target site used to assess off-target methylation. The non-target site lacks HS1 and HS2 zinc finger binding sites, but contains a CpG site nested within an SnaBI restriction site (E) An overview of the selections used in this experiment. The schematic illustrates the fates of plasmids encoding inactive methyltransferase (digested by FspI, left), a desired targeting methyltransferase methylated at the target site (not digested, middle) and a nonspecific methyltransferase methylating multiple M.SssI (i.e CpG) sites (digested by McrBC, right).

An NNK cassette mutagenesis library of M.SssI [273–386] was constructed by overlap extension PCR. PCR was carried out using an oligonucleotide degenerate for a five amino acid region in the C-terminal fragment corresponding to amino acids 297–301 in the wild type enzyme. Fragments were digested with AgeI-HF and SpeI and ligated into pDIMN8 containing HS2 and the complete N-terminal fragment-HS1 fusion (Fig. 1 A,B). HS1 and HS2 have been described previously [33]. Site 1 contained a target site comprised of CpG site nested within an FspI restriction site and flanked by HS1 and HS2 zinc finger recognition sequences (Fig. 1C). The plasmid also possessed a non-target site that lacked zinc finger binding sites but contained an internal SnaBI restriction site (Fig. 1D). Ligations were transformed into ER2267 electrocompetent cells, which were plated onto agarose plates containing 100 µg/ml ampicillin and 2% w/v glucose. Plates were incubated overnight at 37°C. The naive library contained 2×10^5^ transformants.

### Library Selection

Plated library variants were recovered from the plate in lysogeny broth supplemented with 15% v/v glycerol and 2% w/v glucose and stored at −80°C. Aliquots were thawed and used to inoculate 10 ml of lysogeny broth supplemented with 100 µg/ml ampicillin salt, 0.2% w/v glucose, 1 mM IPTG, and 0.0167% w/v arabinose. These cultures were incubated overnight at 37°C and 250 rpm. Plasmid DNA was isolated via QIAprep Spin Miniprep Kit and digested for 3 hours at 37°C with McrBC (10 units/µg DNA), FspI (2.5–5 units/µg DNA) in 1X NEBuffer 2 supplemented with 100 µg/ml BSA and 1 mM GTP. Reactions were halted by incubation at 65°C for over 20 min to which ExoIII (30 units/µg DNA) was added and the solution incubated at 37°C for 60 min. ExoIII digestion was halted by incubation at 80°C for over 30 min and the DNA was desalted using Zymo Clean and Concentrator-5 kits per manufacturer’s instructions. DNA was transformed into ER2267 electrocompetent cells and plated on agar supplemented with 2% w/v glucose and 100 µg/ml ampicillin salt.

Cells were recovered from the plate as before and plasmid DNA was isolated using the QIAprep Spin Miniprep Kit. The DNA was digested with FspI (2–2.8 units/µg DNA) in 1X NEBuffer 4 and linear DNA was isolated via gel electrophoresis. PCR was used to amplify the portion of the linear plasmid containing genes encoding for the N-terminal and C-terminal fragments fused to zinc fingers. Purified PCR products were subcloned into the selection plasmid for an additional round of selection.

### Restriction Endonuclease Protection Assays

Cultures from colonies were incubated overnight at 37°C and 250 rpm in lysogeny broth supplemented with 0.2% w/v glucose and 100 µg/ml ampicillin salt and stored as glycerol stocks. Glycerol stocks were used to inoculate 10 ml of lysogeny broth supplemented with 100 µg/ml ampicillin salt, 0.2% w/v glucose, 1 mM IPTG, and 0.0167% w/v arabinose. After growth overnight at 37°C and 250 rpm, plasmid DNA was purified from the cultures with a QIAprep Spin Miniprep Kit. Plasmid DNA (500 ng) was digested with NcoI-HF (10 units) and either FspI (2.5 units) or SnaBI (2.5 units) in 1X NEBuffer 4 for over one hour at 37°C. SnaBI digests were supplemented with 100 µg/ml BSA. Half of each digested sample was loaded onto agarose gels (1.2% w/v in TAE) and electrophoresed at 90 V for 105–120 minutes.

### Bisulfite Analysis

Glycerol stocks of ER2267 cells containing a plasmid encoding the methyltransferase variants were used to inoculate 10 ml of lysogeny broth supplemented with 100 µg/ml ampicillin salt, 0.2% w/v glucose, 1 mM IPTG, and 0.0167% w/v arabinose. Cultures were incubated for 12–14 hours at 37°C and 250 rpm and plasmids were isolated as above. Plasmids (2 µg) were linearized with 1X NcoI-HF (20 Units/ug DNA) in 1X CutSmart Buffer. Linear plasmids were purified using DNA Clean & Concentrator-5 (Zymo Research, Irvine, CA). Linearized plasmids (500 ng) were treated with bisulfite reagent using the EZ-DNA Methylation Gold Kit (Zymo Research, Irvine, CA). Touchdown PCR, using PfuTurbo Cx Hotstart DNA polymerase was used to amplify regions encoding the target and the non-target sites and was modified from [34]. An initial cycle of 95°C for 3 min was followed by a touchdown PCR (95°C for 1 min, annealing temperature for 1 minute, 72°C for 2 minute). The annealing temperature started at 64°C and was dropped 2°C degrees after two cycles and then decreased 1°C after every other cycle until the annealing temperature reached 52°C. After the touchdown PCR, an additional 30 cycles were carried out with the parameters above and an annealing temperature of 51°C. A final extension was carried out at 72°C for 10 min. The antisense strand at the target site was amplified with primers 5′-AAG ACA GAG CTC AAA CTA AAT AAC CTT CCC CAT TAT AAT TCT TCT’(Fw) and 5′-CCG TAG CCA TGG TAT ATT TTT AAT AAA TTT TTT AGG GAA ATA GGT TAG GTT TTT AT-3′ (Rev). The antisense strand at the non-target site was amplified with primers 5′-AAG ACA GAG CTC CTC TAC TAA TCC TAT TAC CAA TAA CTA CTA CCA ATA A-3′(Fw) and 5′-CCG TAG CCA TGG GTA AAG TTT GGG GTG TTT AAT GAG TGA GTT AAT TTA TAT TAA TTG-3′ (Rev). PCR amplified products were purified by gel electrophoresis as above digested with SacI-HF and NcoI-HF, ligated into pDIMN9 and transformed into NEB 5-alpha Competent *E. coli* (High Efficiency). Individual colonies were sequenced and analyzed using quantification tool for methylation analysis (QUMA) [35]. Low quality sequences were excluded if they had more than five unconverted CpH sites or if less than 95% of all CpH sites were converted. Sequences were also excluded if they either had over 10 alignment mismatches or less than 90% percent identity to the reference sequence.

## Results and Discussion

### Design of the Selection System

Our *in vitro* selection system preferentially enriches variants from a mutagenesis library that possess the ability to methylate a target site, but also lack the ability to methylate other non-targeted M.SssI sites on the plasmid. *In vitro* selection strategies have been used to enrich for methyltransferases with relaxed or altered specificity. Most strategies rely on methylation-dependent protection from restriction endonuclease digestion to positively select for DNA encoding a methyltransferase with altered specificity [36]–[40]. Our selection scheme differs from previous studies as it additionally employs McrBC as a negative selection against unwanted methylation activity. In our system for altering methyltransferase specificity, a single plasmid contains both genes encoding the zinc finger-fused M.SssI fragments as well as a targeted M.SssI CpG site that is nested within an FspI restriction site and flanked by zinc finger binding sequences (Fig. 1A–C). The plasmid also has over 400 other M.SssI (i.e. CpG) sites and a non-target site, comprised of a SnaBI restriction site, for the assessment of off-target methylation (Fig. 1D). Once transformed into *E. coli*, the methyltransferase fragments encoded by the plasmid are expressed, resulting in methylation of the same plasmid. The plasmid DNA is isolated and subjected to *in vitro* digestions with endonucleases FspI and McrBC (Fig. 1E). Since FspI digestion is blocked by methylation, FspI digestion serves to select for methylation at the targeted CpG site. McrBC is an endonuclease that recognizes and cleaves DNA with two distally methylated sites [41], [42]. McrBC will not digest a single site that is methylated or hemimethylated unless there is a second methylated site on the same DNA within about 40–3000 bp [43]. We therefore expect that most plasmids methylated at multiple M.SssI sites will be digested by McrBC. Thus, McrBC digestion selects against off-target methylation. The DNA is then incubated with ExoIII to degrade any plasmid that is digested at least once, ideally leaving the plasmid DNA encoding a highly specific methyltransferase intact for the subsequent transformation.

Initial proof of principal selections demonstrated that McrBC, FspI and ExoIII treatment of unmethylated plasmid DNA, followed by transformation resulted in a 99.85% decrease in the number of transformants relative to untreated DNA. Similarly, McrBC, FspI and ExoIII treatment of a highly methylated plasmid reduced transformants by 99.95% relative to untreated control.

### Design of the Library

We constructed a library of M.SssI C-terminal fragment variants randomized at residues 297–301(Fig. 1B). We hypothesized that mutations to these residues might reduce the ability of the split methyltransferase to methylate non-targeted CpG sites by reducing the fragment’s inherent affinity for double-stranded DNA. Early studies indicated that M.SssI interacts with DNA, irrespective of the presence of CpG sites and subsequently methylates processively [44]. Further, a homology model of M.SssI suggested that residues 297 and 299 form contacts with the ribose phosphate backbone on the CpG bases complementary to the methylated CpG site [45]. Mutational studies showed that for monomeric M.SssI, K297A or N299A mutations did not appreciably affect either the catalytic activity or the dissociation constant of a CpG containing oligonucleotide [46]. Mutating these residues, we hypothesized, might eliminate the innate affinity of our fragments for DNA without affecting the catalytic activity of the enzyme.

Additionally, the homology model indicated the amide backbone of serine residue at position 300 made base-specific contacts with the cytosine and guanine bases complementary to the methylated strand. This model initially implicated serine’s conserved and catalytically important role for stabilizing the complementary strand during base flipping and methylation [45]. However, the S300P mutation resulted in only a three-fold increase in a dissociation constant and no significant change in initial rate of reaction [47].

### Library Selections

Initial selection experiments on this library resulted primarily in the isolation of plasmid DNA with a deleted FspI restriction site, presumably formed by a recombination event. This false positive was a trivial, albeit frequently observed, solution for plasmid survival in our devised scheme. Thus, we subjected the plasmid DNA from the resulting transformants to additional steps to enrich for those plasmids that survived our selection and retained their FspI site. In these additional steps, the plasmid DNA was transformed into ER2267 cells and the cells were plated under conditions known to repress the promoters controlling methyltransferase fragment expression. We digested plasmid DNA from these cells with FspI and purified the linear, FspI-digested DNA away from undigested plasmid DNA by agarose gel electrophoresis. The portion of the plasmid encoding the zinc fingers and methyltransferase genes was PCR amplified, ligated back into the same plasmid backbone, and subjected to an additional round of selection (‘recombinant removal’ step in Fig. 1E). The additional round of selection also included this FspI site-enrichment step. Variants were then randomly selected for further analysis.

### Analysis of Library Variants Identified by Selections

**Figure 2:**
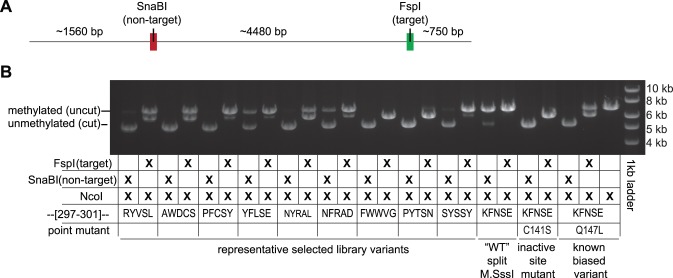
Methylation assay for selected variants. (A) Relative locations of the target site and non-target site on a plasmid linearized by NcoI digestion. For sequences of the target and non-target sites, refer to Figures 1C and D (B) The restriction endonuclease protection assay for methylation at the target and non-target site uses digestion with NcoI to linearize the plasmid and either FspI or SnaBI to assess the target and off-target methylation, respectively. FspI and SnaBI cannot digest a methylated site. Shown are results from select variants as well as the ‘wildtype’ heterodimeric enzyme (i.e. no mutations to residues 297–301) with or without a catalytically inactivating (C141S), or a catalytically compromised (Q147L) mutation.

We assayed 47 variants identified from our selections for methylation activity at both the target and non-target site using our restriction digest assay and determined the variants’ sequences. Representative variants from these digest assays are shown in Figure 2B. Most active variants qualitatively displayed biased methyltransferase activity toward the targeted site. A complete list of sequenced variants can be found in Table S1.

**Figure 3:**
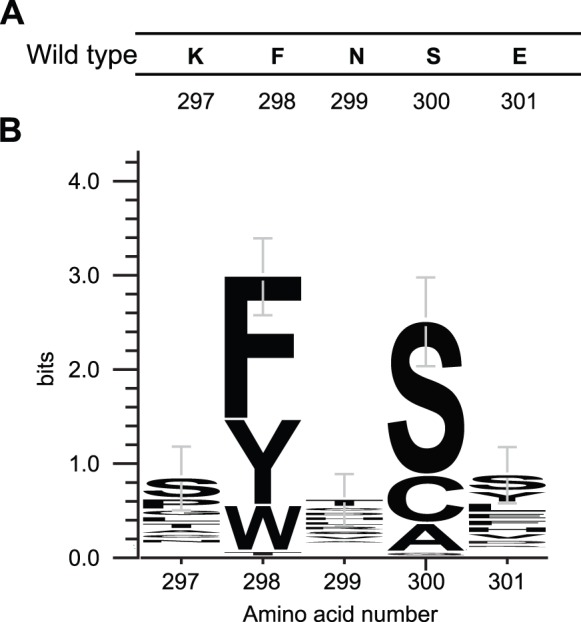
Sequence conservation at residues 297–301 of all catalytically active selected variants. (**A**) The wild type sequence for residues 297–301 (**B**) A sequence logo of active variants.

We compiled a list of amino acid sequences from all the active variants that were assayed in our digestion assays to create a sequence logo using using weblogo 3.3 [48], [49]. This sequence logo indicated that a functional heterodimeric methyltransferase strongly preferred certain residues at positions 298 and 300 (Fig. 3). Position 298 (wildtype phenylalanine) was almost exclusively composed of aromatic residues. Position 300 (wildtype serine) was almost exclusively composed of small residues (defined as an amino acid with an R side chain containing 1–3 heavy atoms). The observed conservation at these residues is consistent with sequence alignments showing these two residues are relatively well-conserved among methyltransferases of different species [45]. In contrast, positions 297, 299 and 301 exhibited little preference for specific amino acids. This finding is consistent with the mutational study discussed above [46]. Our study reveals that there are numerous solutions for improving the specificity of our zinc finger-fused, bifurcated methyltransferases.

**Figure 4:**
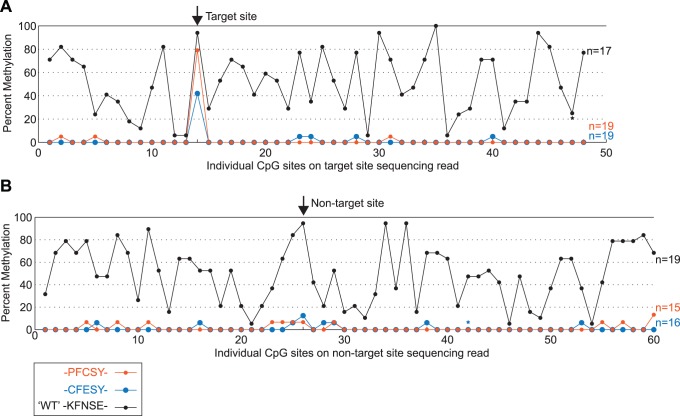
Bisulfite analysis of the frequency of methylation at target, non-target, and other proximal CpG sites. Percent methylation observed at individual CpG sites at and adjacent to the (**A**) target site and (**B**) non-target site. Percentages at each CpG site were determined by bisulfite sequencing of *n* number of clones (*n* indicated at right). CpG sites are numbered sequentially from 1–48 or 1–60 based on their order in the sequencing read and thus, the figure does not indicate the distance between sites. The asterisks indicate two CpG sites at which the methylation status of one clone was judged to be indeterminate due to poor sequencing quality in this region. Black, ‘WT’ heterodimeric enzyme (KFNSE); orange, PFCSY variant; blue, CFESY variant. The target and non-target CpG sites (i.e. the two sites assessed by restriction enzyme digests) are indicated by the arrows. The length of DNA subjected to bisulfite sequencing was 548 bp for the target site and 631 bp for the non-target site.

To further characterize our engineered methyltransferases, we subjected plasmids containing optimized variants, PFCSY, CFESY (named for the sequence at residues 297–301), and the un-optimized ‘WT’ variant to bisulfite analysis at both the target and non-target sites. These plasmids were isolated from cultures grown under conditions known to induce the expression of the plasmids’ methyltransferase fragment fusion genes. In addition to assessing levels of methylation at the target and non-target CpG sites, the regions subjected to bisulfite sequencing assessed the methylation status of 47 and 59 additional CpG sites around the target and non-target sites, respectively (covering over 25% of the total CpG sites present on the plasmid). We sequenced ≥15 clones for each variant to quantify the frequency of methylation at all CpG sequences around both sites (Fig. 4A,B). Based on this sequencing, the PFCSY variant methylated the target site at a frequency of 78.9%. In contrast, only fifteen off-target methylation events were observed in the 34 sequence reads (out of a total of 1793 possible off-target methylation events), which corresponds to an off-target methylation frequency of 0.84%. The PFCSY variant’s specificity for the target site is a marked improvement over the un-optimized, ‘WT’ variant, which methylated the target site at a frequency of 94.1% and off-target sites at a frequency of 49.5%. Thus, for this variant, our selections resulted in the identification of a variant with an almost 60-fold reduction in off-target methylation and a minimal decrease in methylation at the target site. The CFESY variant methylated the target site at a lower frequency compared to the PFCSY variant, but exhibited a similar low frequency of methylation at other CpG sites (target frequency of 42.1% and a 0.71% frequency at all other CpG sites).

### The Targeted Heterodimeric Methyltransferases are Modular

**Figure 5:**
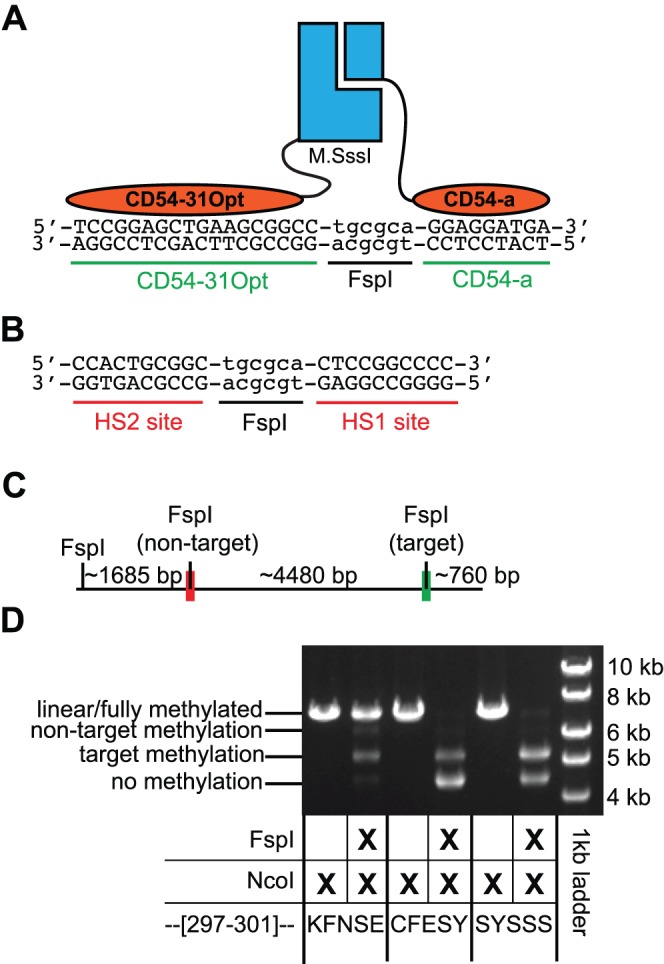
Substitution of new zinc fingers targets methylation towards a new site. (**A**) A schematic of the designed methyltransferase is shown assembled over the new, targeted CpG site. New cognate zinc finger recognition sequences flank a CpG site nested within an FspI site. Zinc fingers CD54-31Opt and CD54a have replaced the HS1 and HS2 zinc fingers. (**B**) The non-target site contains the HS1 and HS2 zinc finger recognition sites flanking a CpG site nested within a FspI restriction site (i.e. this was the target site in experiments in Figure 2). (**C**) The relative locations of the target site and non-target site are shown on a plasmid linearized by NcoI digestion. (**D**) The restriction endonuclease protection assay for methylation at the target and non-target site for the ‘wildtype’ heterodimeric enzyme (KFNSE) and two selected variants with mutations in the region 297–301.

To test whether our targeted M.SssI methyltransferases are modular with respect to the zinc finger domains, we replaced zinc fingers HS1 and HS2 with two zinc fingers designed to target a specific site in the promoter of intercellular adhesion molecule 1 (ICAM1). The previously designed zinc finger CD54-31Opt [50] is adjacent to a CpG site in this promoter. To generate a pair of zinc fingers capable of flanking this CpG site, we designed a second zinc finger, CD54a, to bind downstream from the recognition sequence of CD54-31Opt and adjacent CpG site (Fig. 5A). The two zinc fingers were fused to fragments comprising un-optimized bifurcated M.SssI fragments (residues KFNSE at positions 297–301) and to two selected variants (CFESY and SYSSS at positions 297–301), replacing the HS1 and HS2 zinc fingers (Fig. 5A). These two optimized variants (CFESY and SYSSS) were chosen because preliminary experiments (preformed essentially as described in [17]) suggested that methylation at the target site (containing both zinc finger binding sites) was greater than the additive amount of methylation levels observed at “half-sites” composed of only one or the other of the zinc finger binding sequences.

We assessed the methyltransferase activity and specificity of these constructs in *E. coli* with a restriction endonuclease protection assay at the target and non-target sites (Fig. 5A–D). Notably, the ‘non-target’ site assessed in this experiment contained the zinc finger sequences recognized by HS1 and HS2 zinc fingers (compare Fig. 5B and 1C). Although all three constructs methylated the target site derived from the ICAM1 promoter, the CFESY and SYSSS constructs targeted methylation to the desired site with little to no observable methylation at the non-target site (Fig. 5D).

The CD54-31Opt was chosen because it was shown to effectively target the ICAM1 promoter, altering transcription levels when fused to transcriptional activators or repressors [50], [51]. Additionally, fusion of CD54-31Opt to Ten-Eleven Translocation 2 enzyme resulted in a small, observable amount of demethylation around the target site, correlating with a 2-fold upregulation in ICAM1 transcription [52]. Our construct may potentially enable assessment of the biological affects of targeted methylation at this site.

## Supporting Information

Figure S1 **The DNA and amino acid sequences for the (A) N-terminal and (B) C-terminal M.SssI fragments fused to CD54-31Opt and CD54a respectively.** The methyltransferase fragments (cyan), amino acid linkers (yellow), and zinc finger domains (red) are shown along with the ‘wildtype’ sequence from 297-301 (KFNSE) shown in magenta.
(PDF)

Table S1 **Variants from the selected library.** Sequenced library variants are shown. Aromatic amino acids at position 298 are highlighted in yellow and small amino acids (defined as an amino acid with an R side chain containing 1–3 heavy atoms) at position 300 are highlighted in cyan. Stop codons are denoted by a *. “Assayed” column has an “x” if the variant was tested in the restriction endonuclease protection assay at the target and non-target site. “Active” column has an “x” if the assay indicated protection from restriction enzyme digestion at one or both sites.
(PDF)

## References

[1] Smith ZD, Meissner A (2013) DNA methylation: roles in mammalian development. Nat Rev Genet 14: 204–220 10.1038/nrg3354 23400093

[2] Horsthemke B, Buiting K (2008) Genomic imprinting and imprinting defects in humans. Adv Genet 61: 225–246 10.1016/S0065-2660(07)00008-9 18282508

[3] Bergman Y, Cedar H (2013) DNA methylation dynamics in health and disease. Nat Struct Mol Biol 20: 274–281 10.1038/nsmb.2518 23463312

[4] Xu GL, Bestor TH (1997) Cytosine methylation targetted to pre-determined sequences. Nat Genet 17: 376–378 10.1038/ng1297-376 9398832

[5] McNamara AR, Hurd PJ, Smith AEF, Ford KG (2002) Characterisation of site-biased DNA methyltransferases: specificity, affinity and subsite relationships. Nucleic Acids Res 30: 3818–3830. 12202767 10.1093/nar/gkf501 PMC137423

[6] Carvin CD, Parr RD, Kladde MP (2003) Site-selective in vivo targeting of cytosine-5 DNA methylation by zinc-finger proteins. Nucleic Acids Res 31: 6493–6501. 14602907 10.1093/nar/gkg853 PMC275549

[7] Carvin CD, Dhasarathy A, Friesenhahn LB, Jessen WJ, Kladde MP (2003) Targeted cytosine methylation for in vivo detection of protein-DNA interactions. Proc Natl Acad Sci U S A 100: 7743–7748 10.1073/pnas.1332672100 12808133 PMC164658

[8] Smith AE, Ford KG (2007) Specific targeting of cytosine methylation to DNA sequences in vivo. Nucleic Acids Res 35: 740–754 10.1093/nar/gkl1053 17182629 PMC1807978

[9] Li F, Papworth M, Minczuk M, Rohde C, Zhang Y, et al (2007) Chimeric DNA methyltransferases target DNA methylation to specific DNA sequences and repress expression of target genes. Nucleic Acids Res 35: 100–112 10.1093/nar/gkl1035 17151075 PMC1761428

[10] van der Gun BTF, Maluszynska-Hoffman M, Kiss A, Arendzen AJ, Ruiters MHJ, et al (2010) Targeted DNA methylation by a DNA methyltransferase coupled to a triple helix forming oligonucleotide to down-regulate the epithelial cell adhesion molecule. Bioconjug Chem 21: 1239–1245 10.1021/bc1000388 20593890 PMC2907751

[11] Rivenbark AG, Stolzenburg S, Beltran AS, Yuan X, Rots MG, et al (2012) Epigenetic reprogramming of cancer cells via targeted DNA methylation. Epigenetics 7: 350–360 10.4161/epi.19507 22419067 PMC3368819

[12] Siddique AN, Nunna S, Rajavelu A, Zhang Y, Jurkowska RZ, et al (2013) Targeted methylation and gene silencing of VEGF-A in human cells by using a designed Dnmt3a-Dnmt3L single-chain fusion protein with increased DNA methylation activity. J Mol Biol 425: 479–491 10.1016/j.jmb.2012.11.038 23220192

[13] de Groote ML, Verschure PJ, Rots MG (2012) Epigenetic Editing: targeted rewriting of epigenetic marks to modulate expression of selected target genes. Nucleic Acids Res 40: 10596–10613 10.1093/nar/gks863 23002135 PMC3510492

[14] Nomura W, Barbas CF (2007) In vivo site-specific DNA methylation with a designed sequence-enabled DNA methylase. J Am Chem Soc 129: 8676–8677 10.1021/ja0705588 17583340

[15] Meister GE, Chandrasegaran S, Ostermeier M (2008) An engineered split M.HhaI-zinc finger fusion lacks the intended methyltransferase specificity. Biochem Biophys Res Commun 377: 226–230 10.1016/j.bbrc.2008.09.099 18835252 PMC2586766

[16] Meister GE, Chandrasegaran S, Ostermeier M (2010) Heterodimeric DNA methyltransferases as a platform for creating designer zinc finger methyltransferases for targeted DNA methylation in cells. Nucleic Acids Res 38: 1749–1759 10.1093/nar/gkp1126 20007601 PMC2836561

[17] Chaikind B, Kilambi KP, Gray JJ, Ostermeier M (2012) Targeted DNA methylation using an artificially bisected M.HhaI fused to zinc fingers. PLoS ONE 7: e44852 10.1371/journal.pone.0044852 22984575 PMC3439449

[18] Wicki R, Franz C, Scholl FA, Heizmann CW, Schäfer BW (1997) Repression of the candidate tumor suppressor gene S100A2 in breast cancer is mediated by site-specific hypermethylation. Cell Calcium 22: 243–254 10.1016/S0143-4160(97)90063-4 9481475

[19] Zhang X, Wu M, Xiao H, Lee M-T, Levin L, et al (2010) Methylation of a single intronic CpG mediates expression silencing of the PMP24 gene in prostate cancer. Prostate 70: 765–776 10.1002/pros.21109 20054818 PMC2857536

[20] Renda M, Baglivo I, Burgess-Beusse B, Esposito S, Fattorusso R, et al (2007) Critical DNA binding interactions of the insulator protein CTCF: a small number of zinc fingers mediate strong binding, and a single finger-DNA interaction controls binding at imprinted loci. J Biol Chem 282: 33336–33345 10.1074/jbc.M706213200 17827499

[21] Lukinavicius G, Lapinaite A, Urbanaviciute G, Gerasimaite R, Klimasauskas S (2012) Engineering the DNA cytosine-5 methyltransferase reaction for sequence-specific labeling of DNA. Nucleic Acids Res 40: 11594–11602 10.1093/nar/gks914 23042683 PMC3526304

[22] Pósfai G, Kim SC, Szilák L, Kovács A, Venetianer P (1991) Complementation by detached parts of GGCC-specific DNA methyltransferases. Nucleic Acids Res 19: 4843–4847. 1923753 10.1093/nar/19.18.4843 PMC328777

[23] Lee KF, Kam KM, Shaw PC (1995) A bacterial methyltransferase M.EcoHK311 requires two proteins for in vitro methylation. Nucleic Acids Res 23: 103–108. 7870574 10.1093/nar/23.1.103 PMC306636

[24] Choe W, Chandrasegaran S, Ostermeier M (2005) Protein fragment complementation in M.HhaI DNA methyltransferase. Biochem Biophys Res Commun 334: 1233–1240 10.1016/j.bbrc.2005.07.017 16040000

[25] Ślaska-Kiss K, Tímár E, Kiss A (2012) Complementation between inactive fragments of SssI DNA methyltransferase. BMC Mol Biol 13: 17 10.1186/1471-2199-13-17 22646482 PMC3404938

[26] Renbaum P, Abrahamove D, Fainsod A, Wilson GG, Rottem S, et al (1990) Cloning, characterization, and expression in Escherichia coli of the gene coding for the CpG DNA methylase from Spiroplasma sp. strain MQ1(M.SssI). Nucleic Acids Res 18: 1145–1152 10.1093/nar/18.5.1145 2181400 PMC330428

[27] Sambrook J, Russell D (2001) Molecular Cloning: A Laboratory Manual. 3rd ed. Argentine J, Irwin N, Janssen KA, Curtis S, Zierler M, et al., editors Cold Spring Harbor, NY: Cold Spring Harbor Laboratory Press. 5.4–5.17; 8.21–8.22 p.

[28] Firnberg E, Ostermeier M (2012) PFunkel: efficient, expansive, user-defined mutagenesis. PLoS ONE 7: e52031 10.1371/journal.pone.0052031 23284860 PMC3524131

[29] Sanjana NE, Cong L, Zhou Y, Cunniff MM, Feng G, et al (2012) A transcription activator-like effector toolbox for genome engineering. Nat Protoc 7: 171–192 10.1038/nprot.2011.431 22222791 PMC3684555

[30] Mandell JG, Barbas CF (2006) Zinc Finger Tools: custom DNA-binding domains for transcription factors and nucleases. Nucleic Acids Res 34: W516–W523 10.1093/nar/gkl209 16845061 PMC1538883

[31] Segal DJ, Dreier B, Beerli RR, Barbas CF (1999) Toward controlling gene expression at will: Selection and design of zinc finger domains recognizing each of the 5“-GNN-3″ DNA target sequences. Proc Natl Acad Sci U S A. 96: 2758–2763 10.1073/pnas.96.6.2758 PMC15842 10077584

[32] Blancafort P, Magnenat L, Barbas CF (2003) Scanning the human genome with combinatorial transcription factor libraries. Nat Biotechnol 21: 269–274 10.1038/nbt794 12592412

[33] Beerli RR, Segal DJ, Dreier B, Barbas CF (1998) Toward controlling gene expression at will: specific regulation of the erbB-2/HER-2 promoter by using polydactyl zinc finger proteins constructed from modular building blocks. Proc Natl Acad Sci U S A 95: 14628–14633. 9843940 10.1073/pnas.95.25.14628 PMC24500

[34] Jolly CJ, Neuberger MS (2001) Somatic hypermutation of immunoglobulin kappa transgenes: Association of mutability with demethylation. Immunol Cell Biol 79: 18–22 10.1046/j.1440-1711.2001.00968.x 11168618

[35] Kumaki Y, Oda M, Okano M (2008) QUMA: quantification tool for methylation analysis. Nucleic Acids Res 36: W170–W175 10.1093/nar/gkn294 18487274 PMC2447804

[36] Cohen HM, Tawfik DS, Griffiths AD (2004) Altering the sequence specificity of HaeIII methyltransferase by directed evolution using in vitro compartmentalization. Protein Eng Des Sel 17: 3–11 10.1093/protein/gzh001 14985532

[37] Tímár E, Groma G, Kiss A, Venetianer P (2004) Changing the recognition specificity of a DNA-methyltransferase by in vitro evolution. Nucleic Acids Res 32: 3898–3903 10.1093/nar/gkh724 15273276 PMC506809

[38] Gerasimaite R, Vilkaitis G, Klimasauskas S (2009) A directed evolution design of a GCG-specific DNA hemimethylase. Nucleic Acids Res 37: 7332–7341 10.1093/nar/gkp772 19783820 PMC2790894

[39] Rockah-Shmuel L, Tawfik DS (2012) Evolutionary transitions to new DNA methyltransferases through target site expansion and shrinkage. Nucleic Acids Res 40: 11627–11637 10.1093/nar/gks944 23074188 PMC3526282

[40] Chahar S, Elsawy H, Ragozin S, Jeltsch A (2010) Changing the DNA recognition specificity of the EcoDam DNA-(adenine-N6)-methyltransferase by directed evolution. J Mol Biol 395: 79–88 10.1016/j.jmb.2009.09.027 19766657

[41] Sutherland E, Coe L, Raleigh EA (1992) McrBC: a multisubunit GTP-dependent restriction endonuclease. J Mol Biol 225: 327–348 10.1016/0022-2836(92)90925-A 1317461

[42] Dryden DT, Murray NE, Rao DN (2001) Nucleoside triphosphate-dependent restriction enzymes. Nucleic Acids Res 29: 3728–3741 10.1093/nar/29.18.3728 11557806 PMC55918

[43] Stewart FJF, Raleigh EAE (1998) Dependence of McrBC cleavage on distance between recognition elements. Biol Chem 379: 611–616. 9628366

[44] Renbaum P, Razin A (1992) Mode of action of the Spiroplasma CpG methylase M.SssI. FEBS Lett 313: 243–247. 1446743 10.1016/0014-5793(92)81201-v

[45] Koudan E, Bujnicki J, Gromova E (2004) Homology modeling of the CG-specific DNA methyltransferase SssI and its complexes with DNA and AdoHcy. J Biomol Struct Dyn 22: 339–345. 15473707 10.1080/07391102.2004.10507005

[46] Darii MV, Cherepanova NA, Subach OM, Kirsanova OV, Raskó T, et al (2009) Mutational analysis of the CG recognizing DNA methyltransferase SssI: Insight into enzyme–DNA interactions. Biochim Biophys Acta 1794: 1654–1662 Available: http://linkinghub.elsevier.com/retrieve/pii/S1570963909001824. 19654054 10.1016/j.bbapap.2009.07.016

[47] Darii MV, Kirsanova OV, Drutsa VL, Kochetkov SN, Gromova ES (2007) Isolation and site-directed mutagenesis of DNA methyltransferase SssI. Mol Biol 41: 110–117 10.1134/S0026893307010153 17380899

[48] Schneider TD, Stephens RM (1990) Sequence logos: a new way to display consensus sequences. Nucleic Acids Res 18: 6097–6100. 2172928 10.1093/nar/18.20.6097 PMC332411

[49] Crooks GE, Hon G, Chandonia J-M, Brenner SE (2004) WebLogo: a sequence logo generator. Genome Res 14: 1188–1190 10.1101/gr.849004 15173120 PMC419797

[50] Magnenat L, Blancafort P, Barbas CF (2004) In vivo selection of combinatorial libraries and designed affinity maturation of polydactyl zinc finger transcription factors for ICAM-1 provides new insights into gene regulation. J Mol Biol 341: 635–649 10.1016/j.jmb.2004.06.030 15288776

[51] de Groote ML, Kazemier HG, Huisman C, van der Gun BTF, Faas MM, et al (2013) Upregulation of endogenous ICAM-1 reduces ovarian cancer cell growth in the absence of immune cells. Int J Cancer 134: 280–290 10.1002/ijc.28375 23832872

[52] Chen H, Kazemier HG, de Groote ML, Ruiters MHJ, Xu G-L, et al (2013) Induced DNA demethylation by targeting Ten-Eleven Translocation 2 to the human ICAM-1 promoter. Nucleic Acids Res 42: 1563–1574 10.1093/nar/gkt1019 24194590 PMC3919596

