# Supplementary material for: Directed Evolution of Improved Zinc Finger Methyltransferases
Source: PLoS One. 2014 May 8;9(5):e96931. doi: 10.1371/journal.pone.0096931 (PMC4014571; doi:10.1371/journal.pone.0096931)
Supplement: Figure S1 — The DNA and amino acid sequences for the (A) N-terminal and (B) C-terminal M.SssI fragments fused to CD54-31Opt and CD54a respectively. The methyltransferase fragments (cyan), amino acid linkers (yellow), and zinc finger domains (red) are shown along with the ‘wildtype’ sequence from 297-301 (KFNSE) shown in magenta. (PDF) [file pone.0096931.s001.pdf]

A.

```
 M   S   K   V   E   N   K   T   K   K   L   R   V   F   E   A   F   A   G   I
ATG AGC AAA GTA GAA AAT AAA ACA AAA AAA CTT AGA GTA TTT GAA GCT TTT GCT GGA ATT

 G   A   Q   R   K   A   L   E   K   V   R   K   D   E   Y   E   I   V   G   L
GGT GCT CAA AGA AAA GCC TTG GAG AAA GTC AGA AAA GAT GAA TAT GAA ATA GTA GGG CTT

 A   E   W   Y   V   P   A   I   V   M   Y   Q   A   I   H   N   N   F   H   T
GCT GAA TGG TAT GTT CCT GCA ATT GTT ATG TAT CAA GCT ATA CAC AAC AAT TTT CAT ACA

 K   L   E   Y   K   S   V   S   R   E   E   M   I   D   Y   L   E   N   K   T
AAG TTG GAG TAT AAA TCA GTT TCT AGA GAA GAA ATG ATT GAC TAT TTG GAA AAT AAA ACA

 L   S   W   N   S   K   N   P   V   S   N   G   Y   W   K   R   K   K   D   D
CTA TCT TGG AAC TCA AAA AAT CCA GTA TCT AAT GGT TAT TGG AAG AGA AAA AAA GAT GAT

 E   L   K   I   I   Y   N   A   I   K   L   S   E   K   E   G   N   I   F   D
GAA CTT AAA ATT ATA TAT AAT GCA ATT AAG TTA TCT GAA AAA GAG GGT AAT ATT TTT GAT

 I   R   D   L   Y   K   R   T   L   K   N   I   D   L   L   T   Y   S   F   P
ATT AGA GAC CTT TAC AAA AGA ACT TTG AAA AAT ATA GAT TTA TTA ACA TAT TCA TTT CCT

 C   Q   D   L   S   Q   Q   G   I   Q   K   G   M   K   R   G   S   G   T   R
TGT CAA GAC TTA TCT CAA CAG GGT ATT CAA AAG GGT ATG AAA AGA GGT TCT GGT ACT AGA

 S   G   L   L   W   E   I   E   R   A   L   D   S   T   E   K   N   D   L   P
TCA GGT CTC TTA TGG GAA ATT GAA AGA GCT TTG GAT TCA ACT GAA AAA AAT GAC TTA CCA

 K   Y   L   L   M   E   N   V   G   A   L   L   H   K   K   N   E   E   E   L
AAA TAC TTG TTA ATG GAA AAT GTA GGG GCT CTT CTT CAC AAG AAG AAT GAA GAA GAA CTA

 N   Q   W   K   Q   K   L   E   S   L   G   Y   Q   N   S   I   E   V   L   N
AAT CAA TGG AAG CAA AAA TTA GAA AGT CTT GGC TAT CAA AAC TCA ATT GAA GTT TTG AAT

 A   A   D   F   G   S   S   Q   A   R   R   R   V   F   M   I   S   T   L   N
GCC GCT GAC TTC GGT TCC TCA CAA GCA AGA AGA AGA GTT TTT ATG ATA TCT ACT TTA AAT

 E   F   V   E   L   P   K   G   D   K   K   P   K   S   I   K   K   V   L   N
GAA TTT GTT GAA CTA CCA AAG GGA GAT AAA AAA CCT AAA AGT ATC AAA AAA GTT TTA AAT

 K   I   V   S   E   K   D   I   L   N   N   L   G   G   G   G   S   G   G   G
AAA ATA GTT TCT GAA AAA GAT ATT TTA AAT AAT TTA GGC GGT GGA gga tcc ggc ggc ggt

 G   S   G   G   G   G   S   C   E   K   P   Y   K   C   P   E   C   G   K   S
ggt AGC ggt gga gga ggc TCT TGC GAG AAA CCG TAC AAA TGT CCG GAG TGC GGT AAG AGC

 F   S   D   C   R   D   L   A   R   H   Q   R   T   H   T   G   E   K   P   Y
TTC AGC GAT TGC CGT GAT CTG GCG CGC CAC CAG CGT ACC CAC ACT GGT GAA AAA CCA TAT

 K   C   P   E   C   G   K   S   F   S   R   S   D   D   L   V   R   H   Q   R
AAA TGC CCT GAA TGT GGT AAA AGC TTC TCT CGT TCT GAT GAC CTG GTC CGT CAT CAG CGC

 T   H   T   G   E   K   P   Y   K   C   P   E   C   G   K   S   F   S   Q   S
ACC CAT ACC GGC GAA AAA CCG TAC AAA TGC CCG GAA TGC GGT AAA TCT TTC AGC CAG TCC

 S   N   L   V   R   H   Q   R   T   H   T   G   E   K   P   Y   K   C   P   E
AGC AAC CTG GTT CGC CAT CAA CGT ACT CAT ACT GGC GAG AAA CCG TAC AAA TGT CCG GAG

 C   G   K   S   F   S   T   S   G   E   L   V   R   H   Q   R   T   H   T   G
TGC GGT AAG AGC TTC AGC ACC TCT GGC GAA CTG GTC CGC CAC CAG CGT ACC CAC ACT GGT

 E   K   P   Y   K   C   P   E   C   G   K   S   F   S   Q   R   A   H   L   E
GAA AAA CCA TAT AAA TGC CCT GAA TGT GGT AAA AGC TTC TCT CAG CGT GCG CAC CTG GAA

 R   H   Q   R   T   H   T   G   E   K   P   Y   K   C   P   E   C   G   K   S
CGT CAT CAG CGT ACC CAT ACC GGC GAA AAA CCG TAC AAA TGC CCG GAA TGC GGT AAA TCT

 F   S   Q   A   G   H   L   A   S   H   Q   R   T   H   T   G   K   K
TTC AGC CAG GCG GGC CAT CTG GCG AGC CAT CAA CGT ACT CAT ACT GGT AAA AAG
```

B.

```
 M   E   K   P   Y   K   C   P   E   C   G   K   S   F   S   Q   A   G   H   L
ATG GAA AAA CCA TAC AAA TGC CCG GAG TGT GGC AAA AGC TTT AGC CAG GCG GGT CAT CTG

 A   S   H   Q   R   T   H   T   G   E   K   P   Y   K   C   P   E   C   G   K
GCG AGC CAT CAG CGT ACG CAC ACT GGC GAA AAA CCT TAC AAG TGC CCG GAA TGT GGC AAG

 S   F   S   Q   R   A   H   L   E   R   H   Q   R   T   H   T   G   E   K   P
TCT TTT TCT CAG CGT GCA CAT CTG GAA CGT CAT CAG CGC ACT CAC ACT GGT GAA AAA CCG

 Y   K   C   P   E   C   G   K   S   F   S   Q   R   A   H   L   E   R   H   Q
TAC AAA TGC CCG GAG TGC GGC AAA TCT TTC TCT CAG CGT GCA CAT CTG GAA CGT CAC CAG

 R   T   H   T   G   E   S   G   G   G   G   S   G   G   G   G   L   K   Y   N
CGT ACC CAC ACC GGT GAA TCC GGA ggc ggt ggt TCC ggc ggt gga gga TTG AAA TAT AAT

 L   T   E   F   K   K   T   K   S   N   I   N   K   A   S   L   I   G   Y   S
TTA ACT GAA TTT AAA AAA ACA AAA TCA AAT ATA AAT AAA GCT TCA CTG ATT GGT TAC AGT

 K   F   N   S   E   G   Y   V   Y   D   P   E   F   T   G   P   T   L   T   A
AAA TTT AAT TCA GAA GGT TAT GTT TAT GAT CCT GAA TTT ACA GGA CCA ACC TTA ACT GCA

 S   G   A   N   S   R   I   K   I   K   D   G   S   N   I   R   K   M   N   S
AGC GGT GCA AAT TCA AGA ATA AAA ATC AAA GAT GGA TCT AAT ATT AGA AAA ATG AAC TCA

 D   E   T   F   L   Y   M   G   F   D   S   Q   D   G   K   R   V   N   E   I
GAC GAA ACT TTC TTA TAT ATG GGG TTT GAT TCA CAA GAT GGA AAA AGA GTA AAT GAA ATT

 E   F   L   T   E   N   Q   K   I   F   V   C   G   N   S   I   S   V   E   V
GAA TTT TTA ACT GAA AAT CAA AAA ATA TTT GTT TGT GGA AAT TCA ATA TCA GTA GAA GTT

 L   E   A   I   I   D   K   I   G   G   *
TTG GAA GCG ATT ATA GAT AAA ATT GGA GGT TAA
```
