# Supplementary material for: Directed Evolution of Improved Zinc Finger Methyltransferases
Source: PLoS One. 2014 May 8;9(5):e96931. doi: 10.1371/journal.pone.0096931 (PMC4014571; doi:10.1371/journal.pone.0096931)
Supplement: Table S1 — Variants from the selected library. Sequenced library variants are shown. Aromatic amino acids at position 298 are highlighted in yellow and small amino acids (defined as an amino acid with an R side chain containing 1–3 heavy atoms) at position 300 are highlighted in cyan. Stop codons are denoted by a *. “Assayed” column has an “x” if the variant was tested in the restriction endonuclease protection assay at the target and non-target site. “Active” column has an “x” if the assay indicated protection from restriction enzyme digestion at one or both sites. (PDF) [file pone.0096931.s002.pdf]

Table S1: Variants from the selected library

Colony #	Amino acid at position K297	F298	N299	S300	E301	assayed	active	notes
1	T	F	T	A	H	x	x	
5	P	Y	C	S	F	x	x	
6	G	W	H	S	Y	x	x	
7	C	F	E	S	Y	x	x	
8	V	F	M	*	L			
9	R	F	D	S	L	x	x	
12	S	F	R	C	D	x	x	
13	Y	L	N	G	I	x		
14	S	W	L	S	S	x	x	
15	C	F	A	S	S	x	x	
16	R	R	I	L	*			
18	L	F	L	S	A	x	x	
21	R	W	A	S	*			
22	S	Y	S	S	S	x	x	
23	K	F	N	S	E	x		WT
24	L	W	N	A	S	x	x	
25	H	F	T	S	S	x	x	
26	G	F	E	S	F	x	x	
29	S	F	T	A	R	x	x	
30	S	F	V	S	T	x	x	
31	K	F	N	S	E			WT
32	S	Y	H	S	V	x	x	
34	G	Y	K	C	R	x	x	
35	P	F	F	C	H	x	x	
37	L	K	C	G	G	x		
41	C	F	A	S	S		x	duplicate
42	L	Y	Y	C	E	x	x	
43	L	W	A	S	L	x	x	
45	S	Y	S	C	Y	x	x	
46	R	Y	V	S	L	x	x	
47	S	Y	A	*	M			
49	L	Y	R	*	E			
50	A	W	D	C	S	x	x	
54	P	F	C	S	Y	x	x	
56	Y	F	L	S	E	x	x	
58	L	F	T	A	Y			
59	N	Y	R	A	L	x	x	
62	P	Y	C	S	F		x	duplicate
63	S	F	R	C	D		x	duplicate
64	N	F	R	A	D	x	x	
66	F	W	W	V	G	x		
67	P	Y	T	S	N	x		
69	S	Y	S	S	Y	x	x	
70	L	*	*	Y	P			

71	T	F	T	A	H		x	duplicate
75	S	Y	H	S	V		x	duplicate
76	P	F	V	S	H	x	x	
79	Q	F	M	S	E	x	x	S296N mutation outside cassette; colony not included in weblogo
88	N	F	P	S	F	x	x	
92	A	W	T	S	V	x	x	
95	S	Y	D	S	L	x	x	
98	T	F	N	C	E	x	x	
99	S	Y	H	S	V		x	duplicate
100	F	W	S	S	Q	x	x	
101	P	F	C	S	Y		x	duplicate on amino acid level; different codon usage than colony 54
102	A	F	D	S	S	x	x	
103	I	Y	L	Q	E	x	x	
105	S	Y	V	S	L	x	x	
107	G	T	P	C	T	x	x	
109	V	F	G	C	P	x	x	
110	P	F	T	S	Y	x	x	
114	T	W	F	S	S	x	x	
116	H	F	T	S	S		x	duplicate

Sequenced library variants are shown. Aromatic amino acids at position 298 are highlighted in yellow and small amino acids at position 300 are highlighted in cyan. Stop codons are denoted by a *. “Assayed” column has an “x” if the variant was tested in the restriction endonuclease protection assay at the target and non-target site. “Active” column has an “x” if the assay indicated protection from restriction enzyme digestion at one or both sites.
